# Seroprevalence of hepatitis A infection in a low endemicity country: a systematic review

**DOI:** 10.1186/1471-2334-5-56

**Published:** 2005-07-07

**Authors:** Ba' Pham, Bernard Duval, Gaston De Serres, Vladimir Gilca, Andrea C Tricco, Jan Ochnio, David W Scheifele

**Affiliations:** 1 BioMedical Data Sciences, GlaxoSmithKline, Ontario, Canada; 2 Chalmers Research Group, Children's Hospital of Eastern Ontario Research Institute, Ontario, Canada; 3 Centre De Recherche du CHUQ, Institut Nationale Sante Publique du Quebec, Quebec, Canada; 4 Centre de Recherche du CHUQ, Quebec, Canada; 5 Vaccine Evaluation Centre, University of British Columbia, British Columbia, Canada

## Abstract

**Background:**

In Canada – a low endemicity country, vaccines for hepatitis A virus (HAV) are currently recommended to individuals at increased risk for infection or its complications. Applying these recommendations is difficult because the epidemiology of HAV infection is poorly defined, complex, and changing. This systematic review aimed to 1) estimate age-specific prevalence of HAV antibody in Canada and 2) evaluate infection-associated risk factors.

**Methods:**

MEDLINE (1966–2005) and EMBASE (1980–2005) were searched to identify relevant studies for the systematic review. Archives for the Canada Diseases Weekly Report (1975–1991) and Canada Communicable Disease Report (1992–2005) were searched for relevant public health reports. Data were abstracted for study and participants' characteristics, age-specific prevalence, and risk factors.

**Results:**

A total of 36 reports describing 34 unique studies were included.

The seroprevalence in Canadian-born children was approximately 1% in ages 8–13, 1–6% in 20–24, 10% in 25–29, 17% in 30–39, and increased subsequently. In age groups below 20 and 20–29, age-specific seroprevalence generally remained constant for studies conducted across geographic areas and over time.

Compared to Canadian-born individuals, subjects born outside Canada were approximately 6 times more likely to be seropositive (relative risk: 5.7 [95% CI 3.6, 9.0]). Travel to high risk areas in individuals aged 20–39 was associated with a significant increase in anti-HAV seropositivity (RR 2.8 [1.4, 5.5]). Compared to heterosexuals, men having sex with men were only at a marginally higher risk (adjusted odds ratio 2.4 [0.9, 6.1]). High risk for seropositivity was also observed for Canadian First Nations and Inuit populations.

**Conclusion:**

Results from the current systematic review show that in this low endemicity country, disease acquisition occurs in adulthood rather than childhood. The burden of disease is high; approximately 1 in 10 Canadians had been infected by ages 24–29. The increase in prevalence in young adults coincides with disease importation and increasing frequency of risk factors, most likely behavioral-related ones.

Gaps in seroprevalence data were identified rendering the application of current immunization recommendations difficult. A nationwide prevalence survey for all Canadians is needed. This is essential to quantify the effectiveness of current recommendations and conduct cost-effectiveness evaluations of alternative immunization programs, if necessary.

## Background

Hepatitis A virus (HAV) is prominent in many areas of the world [1-3]. In North America, infection rates have declined with better hygiene practice and public sanitation but remain heterogeneous across geographic and socioeconomic strata [4-6]. Further decline is possible with HAV vaccines which provide consistent, long-lasting protection and have been available since the mid-1990s [7,8]. In the United States, universal vaccination of children and youth has been in place for about 6 years in high endemicity areas [9], leading to historically low rates nationally in recent years [10]. In Canada, the current national immunization guide recommends HAV vaccines for individuals at increased risk of infection or its complications. The guide also states that a universal immunization program should be considered, but further discussion is needed nationally [11].

Applying the Canadian recommendations is difficult because the epidemiology of HAV infection in Canada is poorly defined, complex, and changing [8]. Reported rates differ substantially by province, gender, and age [12]. The rates show repeated peaks and troughs [13] and the last peak occurred in mid-1990 [12,13]. It was during the subsequent period of decline that vaccines were used as a tool to enhance HAV control [8].

Evaluating the impact of the current recommendations is also difficult. Data are needed to distinguish between a cyclical decline and a further decline associated with the recommendations [10]. Such assessment is important to inform future immunization policies. A combination of timely case-notification data, prevalence data, and risk factor data is required for both the application and evaluation of the recommendations.

Case-notification data is of limited use due to under-detection of sub-clinical infection and under-reporting of confirmed cases [8,14]. A useful means that circumvent these limitations is to measure the prevalence of HAV antibody [15]. Following an acute infection, antibody to HAV develops in virtually every instance, remaining detectable for decades, and providing a reliable marker of past infection. In the United States, countrywide seroprevalence surveys and sentinel surveillance have been conducted to provide insight into HA epidemiology, and to rationalize and evaluate immunization programs [10,15,16]. In Canada, similar surveys exist but are limited in scope and comprehensiveness [17-19]. Consequently, risk factor data are also limited and fragmented [12,17]. The current systematic review aimed to 1) estimate age-specific prevalence of hepatitis A antibody in Canada and 2) evaluate infection-associated risk factors.

## Methods

MEDLINE (Jan. 1966 – Mar. 2005) and EMBASE (Jan.1980 – Mar. 2005) were searched to identify citations of potentially relevant studies for the systematic review (MeSH terms: "hepatitis" exploded AND "Canada" exploded). A study report was included if it contained prevalence data of HAV-antibody (detected through sera or saliva samples, hereafter referred to as seroprevalence) for a Canadian population. Reasons for exclusion were categorized and reported. Citations were screened independently by two reviewers. Full-text study reports from citations deemed relevant by one reviewer were obtained. Archives for the Canada Diseases Weekly Report (Jan. 1975 – Dec. 1991, the last year of reporting) and Canada Communicable Disease Report (Jan. 1992 – Mar. 2005) were also searched for potentially relevant public health reports [20]. Two reviewers independently reviewed both published and public health reports. Discrepancies were resolved through discussion. Back referencing and author searches of all included studies were conducted. Other potentially relevant reports were obtained by contacting HA experts and related public health units.

From the included reports, data were independently abstracted for study and participants' characteristics. Age-specific prevalence of HAV antibody data were extracted for Canadian-born participants, all Canadians including individuals born outside the country, and participants with known risk factors [11]. Seroprevalence estimates and 95% confidence intervals (CI) were derived assuming a binomial distribution for the number of seropositive individuals from the total numbers of tested individuals. Participants who reported receiving the HA vaccine were excluded from the prevalence estimates as they were likely to have vaccine-induced antibody.

If available, adjusted odds ratios (AOR) for seropositivity of both demographics and risk factors were extracted, together with the baseline risk of HAV seropositivity (i.e., population, location and timing of the survey). When the AOR of a variable was not reported, HAV antibody data stratified by the variable were obtained to derive the unadjusted relative risk for seropositivity (URR). Relative risk was used as it is a better risk estimate than the odds-ratio in the range of seroprevalence observed in this systematic review [21]. If appropriate, a random effects model was used to combine URRs across studies, together with an assessment for heterogeneity (i.e., chi-squared test).

Information related to the risk of seropositivity was summarized for the following risk categories [11]: 1) travelers to high endemicity areas; 2) groups with high risk activities such as men who have sex with men (MSM), illicit drug users, and street people; 3) First Nations and Inuit populations; and 4) others (e.g., individuals with chronic hepatitis, household contacts, infected food handlers, etc.).

## Results

### Literature search

**Figure 1:**
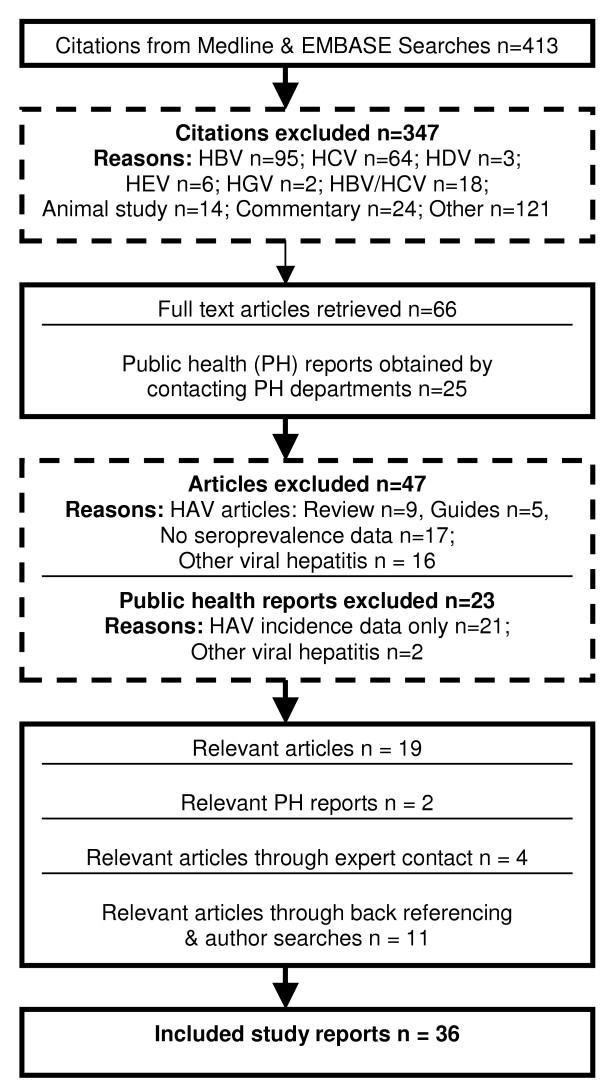
Results of the literature search.

A total of 36 reports describing 34 unique studies were included in the systematic review (Table 1) [18,19,22-55]. These were obtained from screening 413 potentially relevant citations and reviewing 66 full-text study reports and 25 public health reports (Figure 1). Common reasons for exclusion at the screening stage included studies of hepatitis B virus (n = 95), hepatitis C virus (n = 64), commentaries (n = 24), and others (n = 121; Figure 1). Common reasons for exclusion at the full-text review stage included general review of HAV (n = 9), no seroprevalence data (n = 17), and other viral hepatitis (n = 16; Figure 1).

**Table 1:** Study and participant characteristics

**Study**	**Study Design**	**Study Year**	**n**	**Population**	**Location**	**Age in years**
**PUBLISHED LITERATURE**						**Mean ± SD or (range)**

Ochnio 2005* [22]	P	2000–1	811	Grade 9 students	British Columbia	(14–15)
Muecke 2004* [23]	CC	2001	492	Day-care educators	Montreal	37
Minuk 2003 [24]	P	1999	315	First Nations	Manitoba	34 ± 15
Ochnio 2001 [25]	P	1998	494	Street youth, IDU, MSM	Vancouver	19, 35, 34
Smieja 2001 [26]	CC	1997–8	179	IHD patients	Hamilton	61 (38–81)
Kiefer 2000 [27]	R	1997	343	Hepatitis C patients	Edmonton	40 (0–95)
Allard 2001 [28]	P	1995–97	353	Gay men	Montreal	37
Moses 2002 [29]	P	1995–6	533	Street people	Winnipeg	26 (11–65)
Roy 2002 [30]	R	1995–6	427	Street youth	Montreal	(14–25)
Ochnio 1997 [31]	P	1995–6	224	Grade 6 students	Vancouver	(10–12)
De Serres 1997 [32]	P	1995	85	Sewer workers	Quebec	36 ± 7
De Serres 1995 [33]	CC	1995	228	Sewer workers	Quebec	41 (28–64)
Smieja 2003 [34]	R	1993–5	3127	CV or high risk diabetes	Canada	65
Payment 1991 [18]	P	1988–9	617	French-Canadian	Montreal	(9–79)
Embil 1989 [35]	P	1981–3	2036 1922	1/CF recruits 2/CF males	Nova Scotia, Quebec, Posted abroad	1/(15–25) 2/26 (17–53)
Nicolle 1986 [36], Minuk 1985 [37]	P	1982	172	Chesterfield Inlet	Northwest Territories	0 – 78
Crewe 1983 [38]	P	1981–2	304	Children attending outpatient clinic	Halifax	(0.5–16)
Minuk 1982 [39] Minuk 1982 [40]	P	1980	720	Inuit	Northwest Territories	(0.3–86)
McFarlane 1980 [41]	P, R	1980	243 152 293 & 282	1/STD clinic patients 2/Student nurses 3/2 groups of blood donors	Nova Scotia	1/(16–26) 2/(18–24) 3/(16–26) & (51–65)
Buchner 1980 [42]	R	1980	5097	Blood donors	Toronto	<21, >60
Richer 1982 [55]	R	1970–79	447	Samples of acute viral hepatitis	Montreal	Not reported
Minuk 1994 [43]	P	1974–8	42	Household transmission	Winnipeg	27 ± 12
Minuk 2003 [44]	SR	1980–2000	1706	Inuit and First Nations	Various locations	0–60+
McFarlane 1982 [45]	P	NR	154	Institutions	Nova Scotia	(13–28)
McFarlane 1981 [46]	P	NR	130	Patients with hematological malignancy	Nova Scotia	(4–76)

**GREY LITERATURE**						

Duval 2005* [48]	P	2003	1057	Canadian aged 8–13	Canada	(8–13)
Wu 2005 [47]	R	1992–9	NR	Subjects tested for HAV infection	Manitoba	Not reported
Ochnio 2004 [49]	P	2003	585	Young adults	Vancouver	(20–39)
Cook 2000 [19]	R	2000	1206	Women of child-bearing age	British Columbia	(15–44)
Harb 2000 [50]	P	1999	172	First Nations	British Columbia	(0–40+)
Levy 2001 [51]	P	1997	1000	University students	Toronto	25 ± 5
Ford-Jones 1995 [52]	P	1993	122	Day-care providers	Toronto	Not reported
Ochnio 1995 [53]	P	1994–5	1019	Clients to travel clinic	Vancouver	(2–69+)
Kocuipchyk 1995 [54]	P	1991–2	505	Individuals attending travel clinic	Edmonton	(16–60+)

**Notes**:*Study reported seroprevalence data for individuals with or without HAV vaccination.

**Abbreviations: **Study design: P prospective data acquisition, R retrospective data acquisition, CC case control, SR systematic review. Population: IDU injection drug users, MSM men who have sex with men, IHD ischemic heart disease, CV cardiovascular, CF Canadian Forces, STD sexually transmitted disease.

Overall, 74% (n = 25) of the included studies were reported in peer-reviewed journals while 26% (n = 9) were grey literature [20], including 9% (n = 3) public health reports, 14% (n = 5) abstracts, and 3% (n = 1) unpublished study (Table 2). HAV antibody was detected using serum samples in 28 studies and saliva samples in 6. The median sample size was 427 and 793 for the published and grey literature studies, respectively. Only 21% (7/34) of all studies reported prevalence among Canadian-born participants and 29% (10/34) reported prevalence data of all participants including foreign-born individuals. The majority of these studies (27/34) reported prevalence data of participants with known risk factors.

**Table 2:** Study characteristics

	**Published Literature (n = 25)**	**Grey Literature (n = 9)**
	**Peer-reviewed study report (n = 25)**	**Public health report (n = 3)** **Abstract (n = 5)** **Unpublished report (n = 1)**

**Study Design**		
Case-control	2	0
Prospective (P) data acquisition	16	7
Retrospective (R) data acquisition	5	2
P & R data acquisition	1	0
Systematic review	1	0
		
**Sample Size**		
>1000	4	3
100 – 1000	19	6
<100	2	0
Median [1^st^, 3^rd ^Quartile]	427 [224, 720]	793 [422, 1029]
Mean (Min, Max)	877 (42, 5097)	708 (122, 1206)
		
**Timing of data collection**		
2000 – 2004	2	3
1990 – 1999	12	6
1980 – 1989	7	0
1970 – 1979	2	0
Not reported	2	0
		
**Populations with prevalence data**		
Canadian born subjects	4	3
All Canadians¶	6	4
Participants with known risk factors	21	7
		
**Seropositivity test**		
Serum samples	22	6
Saliva samples	3	3

**Notes**: ¶including subjects born outside of Canada

### Age-specific seroprevalence

**Figure 2:**
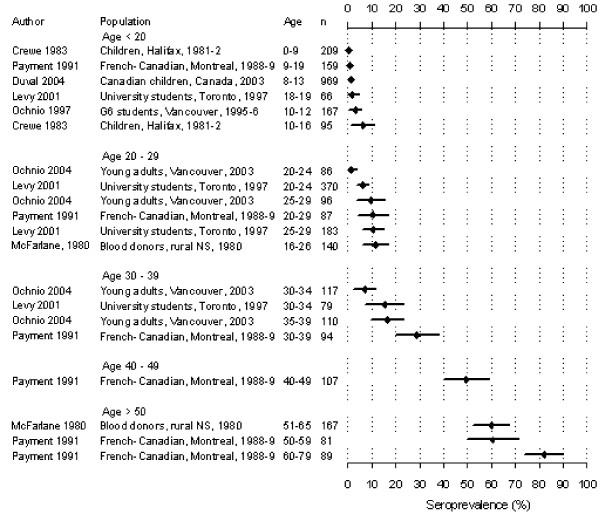
Seropositivity rate (95% confidence interval) among Canadian-born study participants.

The seroprevalence in Canadian-born children aged 8–13 was 1% [95% CI: 0.5–2%] according to a national survey conducted in 2003 [48]. The seroprevalence was 1–6% in ages 20–24, approximately 10% in 25–29, 17% in 30–39, and increased subsequently (Figure 2). In age groups below 20 and 20–29, age-specific seroprevalence generally remained constant for studies conducted across geographic areas in 1980, 1988, 1997, and 2003. This remained so despite differences in study methodology.

There was no association between seropositivity and gender based on 9 population comparisons from 5 studies (n = 4158, URR: 1.0 [95% CI: 0.9, 1.1]) [22,31,35,41,54], which was consistent with results of 3 other studies reporting adjusted risk estimates (Table 3) [25,34,48]. Two studies in the early 1980's suggested that individuals living in urban areas were 30% more likely to have HAV antibody compared to those in rural areas (n = 647, URR: 1.3 [1.2, 1.5]) [41,46].

**Table 3:** Assessment of risk factors

**Risk factor**	**n**	**Risk Measure**	**Risk Estimate (95% CI)**	**Population, Location, Timing of Data Acquisition**	**Age in years**	**Study**
**DEMOGRAPHICS**					**Mean ± SD or (range)**	

Female versus Male	1003	AOR	2.2 (0.8, 6.25)	School-aged children, Canada, 2003	(8–13)	[48]
Female versus Male	3128	AOR	0.8 (0.6, 0.96)	CV or high risk diabetes, Canada, 1993–5	65	[34]
Female versus Male	494	AOR	1.3 (0.8, 2.3)	SY, MSM, IDU, Vancouver, 1998	32 ± 11	[25]
**Female versus Male**	**4158**	**URR**	1.0 (0.9. 1.1) [p = 0.30]*	**9 population comparisons from 5 studies**	**(8–65+)**	[31,35,41,49,54]
**Urban versus Rural**	**647**	**URR**	1.3 (1.2, 1.5) [p = 0.59]*	**3 population comparisons from 2 studies**	(16–76)	[41,46]
Born in high risk country versus born in Canada	494	AOR	2.9 (1.1, 7.6)	SY, MSM, IDU, Vancouver, 1998	32 ± 11	[25]
Born in endemic country versus born in Canada	1003	AOR	22.3 (6.6, 75.0)	School-aged children, Canada, 2003	(8–13)	[48]
Foreign-born versus Canadian-born	353	AOR	6.2 (2.6, 15.0)	Gay men, Montreal, 1995–97	36	[28]
Born in a high-income country versus moderate to low†	492	AOR	20.8 (9.4, 46.0)	Day-care educators, Montreal, 2001	37	[23]
**Foreign-born versus Canadian-born**	**3008**	**URR**	5.7 (3.6, 9.0) [p < 0.01]*	**5 population comparisons from 5 studies**	**(2–69+)**	[30,31,49,51,53]

**TRAVEL TO HIGH RISK AREA**						

Travel to high risk area versus otherwise	1003	AOR	1.4 (0.4, 4.8)	School-aged children, Canada, 2003	(8–13)	[48]
Travel to high risk areas versus otherwise	407	URR	2.8 (1.4, 5.5)	Canadian-born adults, Vancouver, 2003	(20–39)	[49]
Ever travelled to a developing country‡	492	AOR	2.4 (1.3, 4.2)	Day-care educators, Montreal, 2001	37	[23]

**HIGH RISK ACTIVITIES**						

MSM versus heterosexuals	494	AOR	2.4 (0.9, 6.1)	SY, MSM, IDU, Vancouver, 1998	(25–34)	[25]
Sexual partners with VH history versus otherwise	420	AOR	13.8 (4.2, 45.2)	Street youths, Montreal, 1995–6	(14–25)	[30]
Insertive anal penetration versus otherwise	420	AOR	5.1 (1.6, 16.7)	Street youths, Montreal, 1995–6	(14–25)	[30]
History of STD versus no history	500	AOR	2.0 (1.2, 3.3)	Street people, Winnipeg, 1995–6	26 (11–65)	[29]
History of IDU versus no history	494	AOR	6.5 (1.6, 26.3)	SY, MSM, IDU, Vancouver, 1998	(25–34)	[25]
History of IDU versus no history	500	AOR	1.6 (0.99, 2.7)	Street people, Winnipeg, 1995–6	26 (11–65)	[29]

**FIRST NATIONS AND INUIT**						

Native versus Non-native	1003	AOR	5.2 (1.0, 26.0)	School-aged children, Canada, 2003	(8–13)	[48]
Aboriginal versus Non-aboriginal	500	AOR	6.6 (3.8, 11.5)	Street people, Winnipeg, 1995–6	26 (11–65)	[29]
Inuit versus white in NWT	708	URR	4.5 (2.4, 8.5)	Inuits, Baker Lake, NWT, 1980	(0.3–86)	[39,40]
4+ versus 1–3 household occupants	635	URR	1.1 (0.98, 1.3)	Canadian Inuit, Baker Lake, 1980	(0.3–86)	[39,40]

**OTHERS**						

Years working in day-care, 5-year groups§	339	AOR	1.3 (1.0, 1.8)	Canadian-born day-care educators, Montreal, 2001	34	[23]
**History of daycare versus no history**	**1278**	**URR**	1.2 [0.7, 2.2] [p = 0.30]*	**2 population comparisons from 2 studies**	**8–13**	[31,48]
Sewer workers versus controls||	228	URR	1.1 (0.8, 1.4)	Sewer workers, Quebec City, 1993	41 (28–64)	[33]
3+ versus 0–3 siblings	502	URR	2.0 (1.7, 2.5)	Travel clinic, Edmonton, 1991–2	(16–60+)	[54]
Current household income <20,000/yr¶	153	AOR	5.3 (1.2, 24.2)	Foreign-born day-care educators, Montreal, 2001	39.7	[23]
Annual family income <30,000 vs ≥30,000	1057	URR	0.7 (0.3, 2.0)	School-aged children, Canada, 2003	(8–13)	[48]

**Abbreviations: **AOR adjusted odds ratio. CV cardiovascular. SY street youth. MSM men who have sex with men. IDU injectable drug user. URR unadjusted relative risk. STD sexually transmitted disease. NWT North West Territory. G6 grade 6. yr year

**Notes**: *Meta-analytical estimates (95% CI) [p-value from a test of homogeneity] from random-effects models. † The day-care educator study included 492 participants, including 339 Canadian-born individuals and 153 foreign-born. The odds-ratio for "born in a high-income country versus moderate to low" was 20.8 (95% CI 9.4, 46.0) for all 492 participants, not reported for Canadian-born, and 4.6 (1.7, 12.2) for foreign-born. ‡ The odds-ratio for "ever traveled to a developing country" in the day-care educators study was 2.4 (1.3, 4.2) for all 492 participants, not significant for Canadian-born (estimated OR not available), and 8.1 (2.3, 29.0) for foreign-born. § The odds-ratio for "years working in day-care, 5-year groups" was not significant for all 492 participants (estimated OR not available), 1.3 (1.0, 1.8) for Canadian-born, and not significant (estimated OR not available) for foreign-born. || Control subjects were outpatients undergoing lipid testing,. ¶ The odds-ratio for "current household income <20,000/yr" in the day-care educators study was not significant (estimated OR not available) for all 492 participants and Canadian-born and was 5.3 (1.2, 24.2) for foreign-born.

Compared to Canadian-born individuals, subjects born outside Canada were approximately 6 times more likely to be seropositive (n = 3008, URR: 5.7 [3.6, 9.0], Table 3) [30,31,49,51,53], which most likely occurred in their birth country. However, the possibility that infection occurred in Canada could not be ruled out. Age-specific seroprevalence estimates including these individuals varied substantially and could only be used to infer the level of immunity in the population. For example, seroprevalence among all Canadians aged <20 ranged from 2–16% [18,31,38,48,51] for which 3–25% of the sampled populations were individuals born outside the country [25,48,53]. This contrasted the 1% seropositivity for Canadian-born participants reported above.

### Risk factors

Travel-related data were available in 6 studies (Table 1) [23,35,48,49,53,54]. HAV antibody prevalence for Canadian-born individuals visiting a travel clinic was 2.3% in ages 20–25 and 4.3% in ages 25–28 [53]. The prevalence of seropositivity in these individuals was comparable to that reported above for Canadian-born individuals. The risk related to travel among Canadian-born was also not significant in a study of day-care educators [23]. Two population-based surveys reported travel-related risk [48,49]. In one study, travel to high risk areas by Canadian-born individuals aged 20–39 (approximately 12% of study participants) was associated with a significant increase in seropositivity (n = 407, URR: 2.8 [1.4, 5.5]) [49]. In a national survey of children aged 8–13, the prevalence was 1.9% in Canadian-born non-vaccinated travelers and 1.3% in non-travelers; the association was again not significant (Table 3) [48].

Two studies evaluated HAV infection among MSM in two different cities [25,28]. MSM participants on average had 3 sexual partners over the preceding 6 months, according to one study [28]. Also, 18% of these individuals were food handlers. Compared to heterosexuals, MSM were only at a marginally higher risk for seropositivity (n = 494, AOR 2.4 [0.9, 6.1]), according to the second study [25]. However, the study sample was highly heterogeneous and included MSM, injection drug users (IDU), and street youth.

Data on street-involved populations were available in three studies (Table 3) [25,29,30]. Seropositivity was approximately 5% in street youth aged 14–25 in Vancouver and Montreal [25,30]. In the Montreal study [30], the outbreak in MSM (n = 376 cases from December 1994 to February 1998 [28]) seemed to have little effect on the prevalence of anti-HAV among street youth, measured during the same period. Significant behavioral risk factors for seropositivity were reported for street-involved individuals. These included IDU, history of sexually-transmitted disease, and high HAV-risk sexual activities (Table 3).

Seroprevalence in Canadian First Nations and Inuit populations were reported in four studies and summarized in a systematic review [44]. The prevalence ranged from 75–95% and was approximately three times that of non-Aboriginal Canadians residing in the same communities across all ages [44]. For example, Minuk and colleagues reported on a seroprevalence survey of 720 inhabitants of an Inuit community (n = 850). Approximately 27% of this community were aged 0–9, 30% aged 10–19, 32% aged 20–49, and 11% aged 50 or above [40].

Among Canadian-born day-care educators, there was a borderline significant association between risk of HAV positivity and years of employment (Table 3) [23]. A history of daycare attendance among grade 6 students was not associated with seropositivity [31]. Also, the seropositivity was 1.3–1.6 times higher in children aged 8–13 who attended day-care, but no statistical difference was evident in all participants, non-vaccinated participants, or those without known risk factors [48].

Other potential risk factors were also examined. From a sample of 343 individuals who tested positive for hepatitis C, 30% of those aged 20–29 were seropositive for HAV [27]. One prospective cohort followed 62 household contacts and 20 index cases over 6 months; the risk of infection was 52% among other susceptible household members [43]. Working in a sewage plant was not associated with seropositivity [33].

## Discussion

The seroprevalence data consolidated in this systematic review had many limitations. Except for one national survey in ages 8–13 [48], other studies were generally not representative of the general population. Substantial variation across studies was observed with respect to study population, timing, sample size, and location. Reporting of data was inconsistent with respect to age stratification and definition of risk factors. Some studies conducted after the introduction of the vaccine around 1997 did not take vaccine-induced HAV antibody into account. This was, however, rectified in more recent studies [23,48,49]. For example, the seroprevalence in a national survey of children aged 8–13 was 2.7% overall and 2.0% after the exclusion of self-reported vaccinees [48]. The corresponding figures in a survey of young adults aged 20–39 were 22% and 16%, respectively [49]. Given these limitations, improvements in the reporting of future HAV prevalence studies are required. Most importantly, prevalence data should be stratified by participants' birthplace and account for vaccine-induced antibody.

Results from the current systematic review show that disease acquisition occurs in adulthood rather than childhood [14]. In Canada, the increase in prevalence in young adults coincides with disease importation and increasing frequency of behavioral risk factors, such as risk activities among MSM and street-involved populations. Even in this low endemicity country, approximately 1 in 10 Canadians had been infected by ages 24–29.

A low level of HAV immunity in Canada is evident from this systematic review. Over 90% of Canadian-born individuals aged 20–29, and over 80% of those aged 30–39 remained unprotected. Canadians born outside the country generally have a higher prevalence of HAV antibody, yet including these individuals did not significantly improve the percentage of protected Canadians. This low level of immunity and persistent risk of exposure to HAV suggest that outbreaks are possible in the future [14,47]. For example, unprotected clients exposed to an infected food handler led to mass immunizations in the early 2000's (Toronto 2002 [56], n = 19,208; London 2002 [57], n = 16,320; Vancouver 2002 [58] n = 6,000).

Clarifications are required to better understand the epidemiology of HAV in Canada, especially the inter-relation between timely case-notification data, seroprevalence data, and risk factor data. In a study examining national case-notification data from 1990–1999, estimated incidence of reported cases decreased while the average age of exposure and subsequent infection increased [12]. Given the low seroprevalence in Canadian youth, the current results suggest that the average age of HA exposure is above 24 and is increasing. While infection in children is often sub-clinical or mild, infected adults often experience more severe symptoms [1,59].

Results of the current systematic review are consistent with low HAV endemicity patterns in developed countries [60-62]. A substantial burden of infection was observed in young Canadians and this did not decrease among successive generations over the past 20 years. Similar observations were reported elsewhere [63]. In these low endemicity countries, outbreaks are common [64,65]. Sources of outbreaks that are common in these countries include infected food handlers [56,57], contaminated food importation [66,67], and unprotected immigrants who visit friends and relatives in their original countries [68].

In order to apply the current immunization recommendations, substantial information pertaining to groups at increased risk of HA infection or its complications is required. Results from this systematic review suggest that the risk of HA infection in these target groups was not well documented. With the exception of a few population-based surveys [48,49], most studies enrolled participants with known risk factors and failed to include a control group. In addition, some used residual sera obtained for other tests with virtually no risk factor data.

## Conclusion

Results from the current systematic review show that in this low endemicity country, disease acquisition occurs in adulthood rather than childhood. The burden of disease is high; approximately 1 in 10 Canadians had been infected by ages 24–29. The increase in prevalence in young adults coincides with disease importation and increasing frequency of risk factors, most likely behavioral-related ones.

Gaps in seroprevalence data were also identified in this systematic review, rendering the application of current recommendations difficult. A nationwide prevalence survey for all Canadians is needed. This is essential to quantify the effectiveness of current recommendations [10] and conduct cost-effectiveness evaluations of alternative immunization programs, if necessary [69].

## Abbreviations

G6 students grade 6 students

## Competing interests

Funding for this systematic review was provided by GlaxoSmithKline Canada. BP and ACT are employed by GlaxoSmithKline. BD, GDS, VG, JO, and DS have received research funding from GlaxoSmithKline.

## Authors' contributions

BP contributed to the development of the research question and methodology, project management, systematic review, data management and data analysis, derivation of charts and tables, and interpretation of the results. BD, GDS, DS contributed to the development of the research question and methodology, acquisition of unpublished data, and interpretation of the results. JO and VG contributed to the development of the research methodology, acquisition of unpublished data, derivation of charts and tables and interpretation of the results. ACT contributed to the development of the research methodology, project management, systematic review, derivation charts and tables and interpretation of the results. All of us contributed to the manuscript writing and approved the final version of the manuscript.

## Pre-publication history

The pre-publication history for this paper can be accessed here:


